# Health Impacts and Characteristics of Deprescribing Interventions in Older Adults: Protocol for a Systematic Review and Meta-analysis

**DOI:** 10.2196/25200

**Published:** 2021-12-09

**Authors:** Zoë Tremblay, David Mumbere, Danielle Laurin, Caroline Sirois, Daniela Furrer, Lise Poisblaud, Pierre-Hugues Carmichael, Barbara Farrell, André Tourigny, Anik Giguere, Isabelle Vedel, José Morais, Edeltraut Kröger

**Affiliations:** 1 Faculté de pharmacie Université Laval Québec, QC Canada; 2 Centre d'excellence sur le vieillissement de Québec Centre intégré universitaire de santé et de services sociaux de la Capitale Nationale Québec, QC Canada; 3 Faculty of Medicine University of Ottawa Ottawa, ON Canada; 4 Faculté de médecine Université Laval Québec, QC Canada; 5 Faculty of Medicine McGill University Montreal, QC Canada

**Keywords:** deprescribing, older adult, aging, medication use, inappropriate prescribing, potentially inappropriate medication, polypharmacy, comorbidity, multimorbidity, systematic review

## Abstract

**Background:**

Deprescribing, a relatively recent concept, has been proposed as a promising solution to the growing issues of polypharmacy and use of medications of questionable benefit among older adults. However, little is known about the health outcomes of deprescribing interventions.

**Objective:**

This paper presents the protocol of a study that aims to contribute to the knowledge on deprescribing by addressing two specific objectives: (1) describe the impact of deprescribing in adults ≥60 years on health outcomes or quality of life; and (2) determine the characteristics of effective interventions in deprescribing.

**Methods:**

Primary studies targeting three concepts (older adults, deprescribing, and health or quality of life outcomes) will be included in the review. The search will be performed using key international databases (MEDLINE, EMBASE, CINAHL, Ageline, PsycInfo), and a special effort will be made to identify gray literature. Two reviewers will independently screen the articles, extract the information, and evaluate the quality of the selected studies. If methodologically feasible, meta-analyses will be performed for groups of intervention studies reporting on deprescribing interventions for similar medications, used for similar or identical indications, and reporting on similar outcomes (eg, benzodiazepines used against insomnia and studies reporting on quality of sleep or quality of life). Alternatively, the results will be presented in bottom-line statements (objective 1) and a matrix outlining effective interventions (objective 2).

**Results:**

The knowledge synthesis may be limited by the availability of high-quality clinical trials on deprescribing and their outcomes in older adults. Additionally, analyses will likely be affected by studies on the deprescribing of different types of molecules within the same indication (eg, different pharmacological classes and medications to treat hypertension) and different measures of health and quality of life outcomes for the same indication. Nevertheless, we expect the review to identify which deprescribing interventions lead to improved health outcomes among seniors and which of their characteristics contribute to these outcomes.

**Conclusions:**

This systematic review will contribute to a better understanding of the health outcomes of deprescribing interventions among seniors.

**Trial Registration:**

PROSPERO International Prospective Register of Systematic Reviews CRD42015020866; https://www.crd.york.ac.uk/prospero/display_record.php?ID=CRD42015020866

**International Registered Report Identifier (IRRID):**

PRR1-10.2196/25200

## Introduction

### Polypharmacy and Older Adults

The use of multiple medications in older adults is on the rise [1], and the well-known risk of adverse medication events, defined as the undesired effects or toxicities caused by a medication, increases with the number of medications prescribed [2]. Polypharmacy (ie, >5 concurrent medications) among older adults is a worldwide problem with prevalence estimates varying between 39% for the United States in 2010 [3] to 74% in Sweden in 2018 [4]. Associated with aging is the onset of chronic conditions, often coexisting in a phenomenon termed multimorbidity [2]. Polypharmacy may be deemed necessary, or rational, for patients suffering from multimorbidity; this is because, in many cases, at least 1 medication is needed to treat each condition [5]. Polypharmacy is, however, associated with many adverse outcomes, including undesired medication reactions, increased time of hospital stay, as well as the risk of readmission to hospital soon after discharge, falls, and mortality [2]. The risk of an adverse medication event has been estimated at 13% with 2 medications, increasing to 58% and 82% with 5 and 7 or more medications, respectively [6], underlining the need to limit medication use and to prescribe wisely among older and particularly more vulnerable adults.

### Inappropriate Prescribing

Pharmacotherapy is deemed inappropriate when the risks associated with the used medications exceed their benefits [5]. This risk-benefit ratio should therefore be considered by health professionals before prescribing a medication, and it often differs for seniors as they encounter a physiological decline in all aspects of pharmacodynamics and pharmacokinetics [7]. Indeed, potentially inappropriate medication (PIM) can be dangerous for older patients [8]. Several lists of PIMs have been elaborated in an attempt to limit inappropriate prescribing. Explicit criteria, such as the Beers criteria from the United States [9], or the STOPP/START criteria from Europe [10], are widely known and have been adapted to the clinical context of specific countries [11-13]. Health professionals may also use implicit criteria, such as the Medication Appropriateness Index [14]. Given the great number of PIMs and their consequences, medication reviews should be conducted on a regular basis, especially for older adults [5]; however, health care providers still struggle with this process [15].

### Interventions to Improve Inappropriate Prescribing and Polypharmacy

In recent years, interventions to decrease inappropriate prescribing and polypharmacy have increasingly been developed. A Cochrane review [16] and its updates [15,17] studied such interventions: the results from randomized controlled trials (RCTs) on interventions (educational programs for prescribers, medication reviews by pharmacists, and tools to aid in clinical decision-making) showed effectiveness in decreasing inappropriate prescribing, but their clinical effects remained unclear [17].

### Deprescribing

The deprescribing process is defined as the reduction, tapering, or discontinuation of medication deemed inappropriate for a specific patient, with the aim of minimizing polypharmacy and improving patient outcomes [18]. Presently, there is a lack of knowledge regarding the association of specific, effective elements of deprescribing for certain medication classes and the resulting health outcomes. Indeed, knowledge users participating in the development of this study (ie, the National Stakeholder Council on Safe Medication Management for Older Men and Women of the Canadian Institutes of Health research [19], physicians, pharmacists, and patients) have expressed the need for evidence on health outcomes and quality of life regarding deprescribing.

The findings from some RCTs are encouraging: deprescribing of antipsychotics was found to have no detrimental effects [20,21] and to reduce the risk of falls [22]. Deprescribing of benzodiazepines showed subtle cognitive advantages [23], while the discontinuation or dose-reduction of statins in patients with reduced life expectancy had no negative impact [24]. As for the deprescribing of chronic diuretics, 1 study reported preserved health outcomes after deprescribing [25], but 2 others failed to do so [26,27]. A 2016 review by Page et al [28] aimed to determine if deprescribing is a safe, effective, and feasible intervention to reduce mortality in older adults. Their systematic review, reporting on nonrandomized deprescribing interventions, showed a significant decrease in mortality: it found that generalized education programs had no impact on mortality, but patient-specific interventions decreased mortality. Other systematic reviews on the effects of deprescribing of specific medication classes have been performed in the past years (eg, for proton pump inhibitors, benzodiazepines, and antipsychotics [29-31]) in order to develop algorithms to guide deprescribing in clinical practice. Moreover, systematic reviews of the effects of deprescribing among special populations of older adults have been performed, such as a 2018 review by Thillainadasan et al [32] on hospitalized older adults, on older residents in nursing homes by Kua et al [33], in 2019, on older patients with life-limiting illness, by Shrestha et al [34], in 2020, and on older people living with frailty by Ibrahim et al [35], in 2021. Generally, these reviews found deprescribing interventions to be safe and feasible, but they concluded that better evidence on their effects on clinical outcomes was needed; a medication review directed at deprescribing in nursing home residents showed that deprescribing significantly reduced mortality by 26% and the number of fallers by 24%, in a subgroup meta-analysis [35]. Similarly, Hansen et al [36] conducted a systematic review on behavior change techniques in deprescribing and found a combination of such techniques involving a range of interventions to be successful. A 2019 review by Ulley et al [37] found insufficient evidence to confirm that deprescribing improves medication adherence. Bloomfield et al [38] performed a review of the effects of deprescribing interventions on all-cause mortality, hospitalizations, health-related quality of life, and falls among community dwelling older adults in 2020; they concluded that comprehensive medication review may have reduced all-cause mortality, but the certainty of evidence was low, while the effect on hospitalizations, health-related quality of life, and falls was found to be small or absent. Finally, Monteiro et al [39], in a 2019 review, found that computerized decision support tools consistently reduced the number of potentially inappropriate prescriptions started, as well as reducing the mean number of potentially inappropriate prescriptions per patient.

Nevertheless, challenges remain regarding evidence on the health effects of deprescribing interventions across various medication classes and among older adults in particular. To inform about the health outcomes of such deprescribing interventions in older adults, this systematic review will include all recently published intervention studies and report on the characteristics of interventions that are successful at reducing medication and improving or maintaining older adults’ health or quality of life. Ultimately, this systematic review aims to contribute to the development of an interdisciplinary consensus on effective interventions in deprescribing, which may lead to the development of guidance for health professionals and patients, as detailed in the Methods section of the development of public health guidance of the National Institute for Health and Care Excellence [40].

### Objectives

The review will describe the impact of deprescribing on health and quality of life in patients aged 60 years and older, as well as the characteristics of effective deprescribing interventions. To meet these objectives, the study aims to answer the following research questions:

1. What are the impacts of deprescribing interventions in older adults on health outcomes or quality of life?
2. What are the characteristics of deprescribing interventions, or elements thereof, that achieve positive or at least neutral effects on the health or quality of life of older adults?

## Methods

### Literature Review

The review method will be based on the Cochrane Handbook for Systematic Reviews of Interventions [41]. The following keywords and terms will be combined to identify deprescribing intervention studies: (1) polypharmacy, deprescribing, Beers’s criteria, potentially inappropriate; (2) withdraw*, withhold, withheld, stop*, cease*, discontinu*, reduc*; and (3) aged, geriatri*, frai*.

Knowledge users in the study team suggested the collection of relevant key papers that would inform the development of search strategies, which was carried out by 2 scientific librarians in consultation with the review authors. These strategies will be adapted for each database. References will be searched using key international databases such as MEDLINE, EMBASE, CINAHL, Ageline, and PsycInfo, without date limitation. When available, limits will be set to restrict the search to humans aged 60 years and over and publications. Moreover, the search languages will be set to English, French, or German, as the members of the research team are fluent in those languages. The search strategy is featured in Multimedia Appendix 1.

The reference lists of relevant review articles and of included studies will be checked manually for additional relevant articles. We made a special effort to identify relevant gray literature, as can be seen in Multimedia Appendix 2.

### Study Selection

Study selection will be performed by 2 independent reviewers according to the following inclusion and exclusion criteria:

- Population studied: study groups with participants aged 60 years and older will be considered for this review. Study groups with a mean age of ≥60 years, for which at least 80% of participants were ≥60 years old, will be included; we also consider the possibility of extracting data related to a subgroup of participants aged ≥60 years. In addition, the participants will have at least 1 medication prescribed for a chronic condition.
- Interventions: deprescribing interventions, regardless of the intervention target (patients, caregivers, or health professionals) in any intervention setting (hospital, nursing home, etc) will be selected.
- Comparison: only the comparison of deprescribing interventions with usual care or between different types of deprescribing interventions will be considered for this review.
- Outcome: for interventions having affected the participants’ medication regimen, all health outcomes will be considered, including withdrawal symptoms, adverse medication reactions, clinical outcomes, cognition, behavior, falls, use of health services, quality of life, mortality, or survival.
- Study design: all robust study designs will be included (RCT, non-RCT; controlled before-after studies; interrupted time-series studies; and repeated measures).

All included studies will be primary studies; therefore, reviews, editorials, letters to the editor, commentaries, and other similar publications will be excluded.

All identified references will be combined into an EndNote library, and multiple copies will be eliminated. The systematic review software DistillerSR (Evidence Partners) will be used for the subsequent steps. Two reviewers will independently determine the eligibility of the retrieved studies by comparing their titles and abstracts to the inclusion criteria. Subsequently, the full texts of the retained articles will be screened to confirm their relevance. The process will be similar for all types of literature sources. The study selection form can be found in Multimedia Appendix 3.

### Data Extraction

The extraction of data will be completed using DistillerSR (Evidence Partners) and pre-established forms regarding the following:

- Study characteristics (design, date, and location)
- Population selection and participants’ characteristics (age, sex, and residency)
- Intervention description (providers, targets, duration, and follow-up)
- Outcomes (medication regimen, health, and quality of life)

An example of a data extraction form is featured in Multimedia Appendix 4.

The Cochrane Collaboration’s GRADE (Grading of Recommendations, Assessment, Development and Evaluations) approach will be used for grading the quality of the body of evidence for each analyzed intervention outcome [42]. The GRADE score, varying from high to moderate, low, or very low quality, will indicate the level of confidence we have in the effect of the intervention, as reported in any study. The risk of bias of the individual, eligible studies will be assessed using the SIGN (Scottish International Guideline Network) tool for observational studies [43] and the Cochrane risk of bias tool for RCT studies [42]. The process will be carried out by 1 reviewer and reviewed by a second one.

### Data Synthesis

A meeting after the first selection process between researchers and knowledge users (ie, patient experts and clinicians) allowed us to prioritize analyzing the deprescribing of specific medication classes with particular indications (eg, bisphosphonates against osteoporosis) over analyzing the deprescribing effect on one more general health outcome (eg, mortality reduction, as in the review by Page et al [28]). If methodologically feasible, meta-analyses will be performed for intervention studies reporting on deprescribing interventions for similar medications or a specific medication class, used for similar or identical indications, and reporting on similar outcomes (eg, benzodiazepines against insomnia, reporting on sleep quality). If meta-analyses are not possible, the study results will be summarized in a transparent and reproducible narrative synthesis, based on the methods published by Rodgers et al [44,45] (objective 1). Descriptive numerical summary tables will also be completed, including but not limited to the following patient characteristics: (1) author, year, country; (2) study design and setting; (3) number of participants, mean (SD) age, male proportion (%); (4) intervention or control; (5) outcome measures; (6) follow-up duration; and (7) study results (effect of intervention on discontinuation; and health outcomes, quality of life outcomes). In order to answer objective 2, we will identify the most effective interventions or the associated intervention components.

Comparative qualitative analysis will be used to analyze the causal contribution of different intervention components toward health outcomes [46]. The sets of characteristics associated with the specific outcome will be charted. Afterward, they will be subjected to a minimization procedure to identify a simpler set of conditions accounting for observed health outcomes. This will result in a matrix of intervention characteristics and related outcomes.

### Meta-analyses

If outcomes or medications are sufficiently similar, the results of deprescribing interventions will be subjected to meta-analyses using RevMan 5.3.5 (The Nordic Cochrane Centre). Comparisons between interventions will be performed for one outcome at a time. A risk ratio will be estimated for the studies comparing an intervention group with a usual care or control group, using a random effects model, assuming that the risk of publication bias will be low. For studies with more than one intervention group, the usual care or a control group will be appropriately split for each intervention, and a sensitivity analysis will evaluate the impact of this split [47,48]. Any heterogeneity indicated by the χ^2^ test of heterogeneity and the I^2^ statistic with its 95% CI [49] will be investigated through subgroup analyses. Though care will be taken to reduce the risk of publication bias, this assumption will be investigated by a funnel plot analysis [50]. If the follow-ups of interventions have different durations, the effect of the intervention has to be comparable at different times of follow-up. In such a case, a meta-regression model may be used to investigate the potential effect of follow-up duration on the results [51].

### Integrated Knowledge Exchange

The participation of patients and clinical experts in the fields of deprescribing and geriatrics is crucial in order to make the review useful for patients and health professionals, as well as making it applicable to the local setting. Hence, researchers and knowledge users met after the selection of eligible studies and will meet again before the submission of the publication of the results. Each of these meetings will follow a rigorous and transparent methodology and will be documented in detail. The knowledge user team will discuss the prioritization of medication classes for individual review chapters and will also participate in the final interpretation of results before publication, giving their own perspective as clinicians, patients, and decision-makers. All data generated or analyzed during this study will be included in published articles.

## Results

To respond to the first research question, bottom-line statements based on the evidence gathered or the results from meta-analyses will be formulated. For the second research question, a matrix will summarize the effective deprescribing interventions or their components. The results will then be interpreted by the knowledge user team in order to determine their merits and the need for additional research to validate the identified deprescribing interventions. They will also assist the team in determining which results can be generalized for certain medication groups and which require further specific research. The review results will then be modified and finalized through email exchanges.

The review results will be published in peer-reviewed, open-access journals and disseminated to all stakeholders in different forms (eg, as web-based guidance) as part of continuous education material or as documentation for health professionals. They will be integrated into health professionals’ academic training and presented at appropriate annual meetings and websites. The study results (ie, evidence for successful interventions and recommendations to adapt or develop interventions) will be communicated to patients, caregivers, the scientific community, stakeholders, health professionals, and the general community. An integrated knowledge transfer strategy targets this aim (Table 1).

This study is expected to conclude in the winter of 2022.

**Table 1:** End of project knowledge transfer strategy.

Medium	Target
Journal publication	Peer-reviewed, open-access journal (undefined yet)
Communication	Annual meeting of: Canadian Geriatrics Society Canadian Association of Population Therapeutics Family Medicine Forum Le Fonds de recherche du Québec – Santé (FRQS), the Quebec Network for Research on Aging Canadian Pharmacists’ Association (CPhA)
Website publications	Publishing the results on the following websites: Canadian Geriatrics Society Canadian Association of Population Therapeutics Family Medicine Forum FRQS
Web-based guidance and documents	Present successful characteristics of deprescribing intervention on the CPhA website via the initiative “the Translator” The Quebec “Institute National d’Excellence en Santé et en Services Sociaux” will participate in knowledge exchange via the development of guidance documents
Education and training	Offer a continuous education activity for health professionals, led by the Centre d’excellence sur le vieillissement de Québec, which has a strong record of continuous education for clinicians in all settings Integrate into health professionals’ academic training
Continuous collaborations	Pursue our collaboration with several institutions: Seniors Health Research Transfer Network and OPEN (Ontario Pharmacy Evidence Network) Institute for Health Services and Policy Research at the Canadian Institutes of Health Research Canadian Dementia Knowledge Translation Network

## Discussion

### Expected Challenges

Several challenges may be encountered during the review. Preliminary searches have identified some high-quality deprescribing interventions [20-23,26,52], but there may still be a lack of studies describing health outcomes, as noted by Page et al [28]. Their literature search was completed in February 2015, so more study results can be expected, given the increased acceptance of deprescribing and the expressed need for more high-quality deprescribing RCTs [53]. Gray literature was found to be scarce on deprescribing interventions, but trial registers will also be checked to assure complete coverage.

Deprescribing interventions may be available for some medication groups only. We may thus not be able to generalize the evidence on these intervention elements to other medication classes. Given the great variety of studies, it may be difficult to retrieve studies that will be sufficiently homogenous to allow for meta-analyses. Finally, the studies may report various health outcomes, and their relevance could be difficult to compare. To solve this problem, the knowledge user experts will evaluate the importance of such limitations, disregarding certain evidence if deemed not relevant for the clinical context.

Deprescribing interventions for different medication classes, such as psychotropics or statins, may yield nonhomogeneous results, which will be challenging. However, there may exist common characteristics among deprescribing interventions for different medication classes, such as careful patient selection or continuous patient surveillance, leading to positive health outcomes in deprescribing for different medication classes. Finally, different health outcomes may be reported for interventions on similar medications (eg, blood pressure or incidence of cardiovascular disease for the deprescribing of antihypertensives), and it may be challenging to prioritize their relevance.

### Risk of Bias

Some biases, possibly inherent to the review process itself, will be addressed by the review methodology to minimize their effect on the review’s results.

### Selection and Information Bias

Deprescribing was termed in 2003 [54] and only became a Medical Subject Heading term in 2016. Therefore larger, more scoping terms, such as “discontinuation,” will be used for database searches regarding earlier studies on medication discontinuation. We expect this to lead to a large number of retrieved references. Having 2 independent reviewers screen all references is meant to limit selection bias. The team will carefully verify gray literature (Multimedia Appendix 2) to gain the most complete review possible.

### Confounding Bias

Retrieved studies may lack homogeneity, making planned meta-analyses more difficult. Moreover, some health outcomes may not be comparable. We will perform narrative syntheses for medication classes where meta-analyses will be impossible. Furthermore, confounding factors may have not been considered in some studies, affecting the quality grades of these studies and of the resulting evidence. The results of this systematic review will help to identify deprescribing interventions leading to desired health or quality of life outcomes and therefore contribute to a better understanding of how deprescribing may improve seniors’ health and well-being.

## Appendix

Multimedia Appendix 1 Final search strategy.

Multimedia Appendix 2 Complete list of consulted databases and websites.

Multimedia Appendix 3 DistillerSR Deprescription selection questions.

Multimedia Appendix 4 Data extraction grid.

Multimedia Appendix 5 Peer reviewer report from the Canadian Institutes of Health Research.

## Abbreviations

PIM: potentially inappropriate medication
RCT: randomized controlled trial
