# Supplementary material for: Health Impacts and Characteristics of Deprescribing Interventions in Older Adults: Protocol for a Systematic Review and Meta-analysis
Source: JMIR Res Protoc. 2021 Dec 9;10(12):e25200. doi: 10.2196/25200 (PMC8704115; doi:10.2196/25200)
Supplement: Multimedia Appendix 2 [file resprot_v10i12e25200_app2.docx]

**Appendix 2: Complete list of consulted databases and websites^[[1]](#footnote-1)^**

| **Databases** |  | **Websites** |
| --- | --- | --- |
| Evidence-Based medicine (EBM) Reviews |  | Canadian Institutes of Health Research (CIHR) |
| The Cochrane Library |  | Path clinic |
| Campbell Library of Systematic Reviews |  | Health Evidence |
| The Joanna Briggs Institute EBP Database |  | National Institute on Aging |
| MEDLINE (via PubMed) |  | Bruyère continuing care Institute, Ottawa, Canada |
| EMBASE |  | Institut national d’excellence en santé et en services sociaux du Québec (INESSS) |
| Ageline |  |  |
| Cumulative Index of Nursing and Allied Health Literature (CINAHL) |  | Canadian Agency for Drug and Technologies in Health (CADTH) |
| International Pharmaceutical Abstracts |  |  |
| PsycINFO |  |  |
| Centre for Reviews and Dissemination (CRD) |  |  |
| SCOPUS |  |  |
| Health Services research projects in Progress |  |  |
| Germain (Bibliothèque de gériatrie et de gérontologie, IUGM) |  |  |
| GrayLIT Network |  |  |
| GreyNET |  |  |
| Health Services Research projects in Progress |  |  |
| National Information Center on Health Services Research and Health  Care Technology |  |  |
| New York Academy of Medicine Grey Literature Report |  |  |
| OpenGrey and OpenSIGLE |  |  |
| ProQuest Dissertation & Theses |  |  |
| Trial registries (ClinicalTrial.gov, EU Clinical Trials register, International Clinical Trials Registry Platform, Australian New Zealand Clinical Trials Registry, Clinical Trial Registry |  |  |

1. Sources identified *a priori*. Additional databases and websites could be included during the course of the study. [↑](#footnote-ref-1)
