# Supplementary material for: Health Impacts and Characteristics of Deprescribing Interventions in Older Adults: Protocol for a Systematic Review and Meta-analysis
Source: JMIR Res Protoc. 2021 Dec 9;10(12):e25200. doi: 10.2196/25200 (PMC8704115; doi:10.2196/25200)
Supplement: Multimedia Appendix 3 [file resprot_v10i12e25200_app3.docx]

**Appendix 3: DistillerSR Deprescription selection questions**

**Distiller L1: Title screening**

**Distiller L2: Abstract screening**

**Distiller L3 – Full text screening guide**

1. Study participants are older adults.

- Yes, participants are at least 60
- Yes, participants' mean age is at least 60
- Yes, at least 80% of participants are at least 60
- Yes, it seems possible to extract data specific to participants 60+
- No
- Not clear / not reported

2. The study comprises a deprescribing intervention

- Yes
- No
- No, follow-up study

3. The intervention succeeded in switching, reducing or discontinuing the target medication(s)

- Yes
- No
- Not reported
- Not applicable

4. At least one health outcome or one quality of life outcome is reported

- Yes, health outcomes
- Yes, quality of life outcomes
- No

5. Is the report unusable for any of the reasons below?

- No
- Unusable: study on treatment length not really deprescription
- Unusable: results cannot be extracted
- Unusable: incomplete/insufficient information
- Unusable: no primary data
- Unusable: full report could not be retrieved
- Unusable: deprescription is not the main focus of the study (e.g. only mentioned in the discussion or references)
- Unusable: language other than English, French or German
- Unusable: model / simulation
- Yes, unusable
- Short-term interruption
- Clinical trial comparing Rx

6. Comments…

7. Should this reference continue to level 4 (extraction)?

- Yes
- No

**Distiller L4 –** **Quality Control – Study Characteristics**

1. Randomized trial?

- Yes
- No

2. Detailed study design

- Randomized controlled trial (RCT)
- Cluster randomised trial
- Subgroup of a randomized controlled study
- Randomized trial
- Controlled sub-analysis of a randomized intervention
- Cross over study
- Before and after comparison study without control group
- Before and after comparison study with control group
- Interrupted time series
- Cohort study
- Prospective study
- Prospective two-period study
- Observational prospective
- Prospective three-period study
- Retrospective review of database
- Retrospective observational
- Case-control study

3. Study objectives…

Geographic Location

4. Number of sites

- 1 site
- multicentre, specify ____________________

5. Geographical location, principal site

- International United States (specify state)
- Canada (specify province)
- United Kingdom (specify country)
- Australia / New Zealand (specify country)
- Europe (specify country)
- Israel Asia (specify country)
- Africa (specify country)
- Not specified
- Latin America

Study Period

7. Year the recruitement started ____________

8. Year of study end _____________

9. Anything special during that period?

- No, nothing mentionned
- Yes (specify).___________________

Participants

10. Participants recruitment

- Public advertisement (specifiy)
- Through health care provider(s) (specifiy)
- private endocrinology practice
- Through establishment (clinic, hospital, etc.)
- From medical records
- Census tracts, probability samples of larger areas, or entire housing projects
- Not described

11. Eligibility criteria

12. Participants residency

- Community dwelling
- Nursing home (or other long-term care facilities)
- Mixed
- Not described
- hospital
- Outpatients

13. Participants age (years) _______________

14. Age measure

- Mean
- Mean (SD)
- Mean (range)
- Median
- Range

15. Proportion of men/males (%) ____________

16. Should this reference continue to the next form?

- Yes
- No, incomplete
- No

17. Comments…

**Distiller L4 –** **Quality Control – Intervention Description**

1. Intervention type

- Information/Education
- Medication Review
- Geriatricians' services
- Pharmaceutical Interventions
- Multidisciplinary Teams
- Computarized Support Systems
- Multi-faceted approaches
- Regulatory Policies
- Dose reduction
- Tapered discontinuation
- Direct discontinuation
- Relaxation training
- Cognitive behavioral therapy
- Counselling and psychological support
- Treatment according to guidelines
- Not specified
- Discontinuation (tapered or direct not mentionned)
- Administration of another drug

2. To whom is the intervention addressed?

- patient/participant
- caregiver (family)
- general practionner
- specialist physician
- pharmacist
- nurse
- health care professional(s)
- Not clear / not described
- Not applicable

3. Intervention administered by

- Research/study team
- Member of the research/study team (specify)
- Clinical Pharmacist
- Specialist physician
- General Practitioner
- Nurse
- Psychologist
- Clinic staff member (specify)
- Hospital staff member (specify)
- NH staff member (specify)
- Community Pharmacist
- Patient/Participant
- Family member or other non-professional carer
- Not clear / not specified

4. Intervention setting

- Participant's Home
- Family practice
- Day Care Clinic
- Clinic
- Community Pharmacy
- Long-term care facitliy (NH)
- Hospital
- Research setting
- Not clear / not specified

5. Which medication(s) are targeted?

- Polypharmacy
- Inappropriate Medications
- Specific Medication Classe(s)
- Specific Medication

6. Criteria used to identify target medication(s)

- Appropriateness - Beers' criteria
- Appropriateness - STOPP/START criteria
- Appropriateness - Medication Appropriateness Index
- Appropriateness - According to Guidelines
- Appropriateness - Research Team Criteria
- Polypharmacy: 10+
- Polypharmacy: 5+
- adverse drug effects
- Inefficiency
- Effectiveness
- Duration of drug action after discontinuation
- Not described

7. Information regarding the reliability and validity of the criteria provided?

Which one? ­­­­­­­_________________________

- Yes
- No
- Not applicable

Intervention characteristics

8. Intervention duration

- Punctual intervention
- Fixed (number of months)
- Variable (range in months)
- Fixed (number in weeks)
- Variable (range in weeks)
- Not specified

9. Study duration (months)

- Fixed (months) _____________
- Variable (range in months) _____________
- Variable (mean in months) _____________
- Variable (median in months) _____________

10. Subsequent follow-up ?

- Yes (months) _____________
- Yes (weeks) _____________
- No
- Not reported
- Comments _____________

Control group

11. Number of participants in the control group (baseline) _____________

12. What are the main elements of the "non intervention" for the control group? __________

Intervention group

13. Number of participants in the intervention group (baseline) _____________

14. What are the main elements of the intervention for the experimental group(s)? _______

15. Comments ...

16. Should this reference continue to the next level?

- Yes
- Incomplete
- No

**Distiller L4 –** **Quality Control – Main relevant results**

Medication Regimen

1. Describe changes in medication regimen

2. Were changes in health-related outcomes reported?

- Yes, Describe changes in health-related outcomes
- No

Quality of life outcomes

3. Were changes in quality of life outcomes reported?

- Yes, Describe changes in quality of life outcomes
- No

4. Should this reference continue to the next level?

- Yes
- Incomplete
- No

5. Comments ...
