# Supplementary material for: Health Impacts and Characteristics of Deprescribing Interventions in Older Adults: Protocol for a Systematic Review and Meta-analysis
Source: JMIR Res Protoc. 2021 Dec 9;10(12):e25200. doi: 10.2196/25200 (PMC8704115; doi:10.2196/25200)
Supplement: Multimedia Appendix 4 [file resprot_v10i12e25200_app4.docx]

**Data extraction grid**

| # Distiller | Author, year, country | Study design and setting | Number of participants  Mean (SD) age  Male proportion (%) | Intervention/control | Outcome measures | Follow-up duration | Study results | |
| --- | --- | --- | --- | --- | --- | --- | --- | --- |
|  |  |  |  |  |  |  | Effect of intervention on discontinuation | Health outcomes, quality of life outcomes |
|  |  |  |  |  |  |  |  |  |
|  |  |  |  |  |  |  |  |  |
|  |  |  |  |  |  |  |  |  |
