# Supplementary material for: Health Impacts and Characteristics of Deprescribing Interventions in Older Adults: Protocol for a Systematic Review and Meta-analysis
Source: JMIR Res Protoc. 2021 Dec 9;10(12):e25200. doi: 10.2196/25200 (PMC8704115; doi:10.2196/25200)
Supplement: Multimedia Appendix 5 [file resprot_v10i12e25200_app5.pdf]

Canadian Institutes of Health Research / Instituts de recherche en santé du Canada

Notice of Decision / Avis de décision

Application Number/Numéro de la demande : 366106
Committee Code/Code du comité : KRS

Applicants/Candidats :	Docteure Edeltraut Kroger	Dr. José Antonio Morais	
With/Avec :	Madame M. Breton	Dr. B. Farrell	Docteure A. Giguère
	Docteure D. Laurin	Dr. S. Lemire	Docteure M. Morin
	Docteure C. Sirois	Docteur A. Tourigny	Docteure I. Vedel
Research Institution/ Établissement de recherche :	Université Laval		
Institution paid/ Établissement payé :	Université Laval		
Primary Inst./ Inst. principal :	Vieillissement		
Other Related Inst./ Autres inst. connexes :	Services et politiques de la santé; Santé publique et des populations		
Title/Titre :	Health impacts and characteristics of deprescribing interventions in older adults - a systematic review.		

**Decision on your application/ Décision sur votre demande :**	Approved for Funding/ Approuvée pour du financement
**Average annual amount/ Montant annuel moyen :**	$100,000
**Equipment amount/ Montant pour les appareils :**	$0
**Term/Durée :**	1 year(s)/an(s) 0 month(s)/mois

**Competition Outcome/Résultats du concours :**	Knowledge Synthesis Grant/Subvention sur la synthèse des connaissances January/Janvier 25, 2016
**Committee/Comité :**	Subvention sur la Synthèse des Connaissances
**Number of Applications Submitted/Nombre de demandes présentées :**	193
**Number accepted for Final Stage/ Nombre de demandes acceptées à l'étape finale :**	45
**Number Approved for Funding/ Nombre de demandes approuvées pour du financement :**	16

Peer Review Committee Recommendation, for your information and use/
Recommandation de l'évaluation par les pairs, pour fins d'information et d'utilisation :

**Final Application Rank/Classement final de la demande :**	4/193
**Decision at Stage 1/Décision à l'étape 1 :**	Accepted for Final Stage/Acceptée à l'étape finale
**Decision at Final Stage/Décision à l'étape finale :**	Approved for Funding/Approuvée pour du financement
**Recommended Average Amount/Montant annuel moyen recommandé :**	$100,000
**Recommended Equipment Amount/Montant recommandée pour les appareils :**	$0

Consolidated Ranking: The average of all reviewers' individual rankings of the application, expressed as a percentage.
Classement combiné : moyenne des classements individuels de tous les évaluateurs pour une demande, exprimée en pourcentage.

This document is for information only. Official payment is stated on the CIHR Authorization for Funding.
Document à titre d'information seulement. Le paiement officiel est indiqué sur l'autorisation de financement des IRSC.

CIHR IRSC

Institute of Aboriginal Peoples' Health
Institute of Aging
Institute of Cancer Research
Institute of Circulatory and Respiratory Health
Institute of Gender and Health
Institute of Genetics
Institute of Health Services and Policy Research
Institute of Human Development and Child and Youth Health
Institute of Infection and Immunity
Institute of Musculoskeletal Health and Arthritis
Institute of Neurosciences, Mental Health and Addiction
Institute of Nutrition, Metabolism and Diabetes
Institute of Population and Public Health

Institut de la santé des Autochtones
Institut du vieillissement
Institut du cancer
Institut de la santé circulatoire et respiratoire
Institut de la santé des femmes et des hommes
Institut de génétique
Institut des services et des politiques de la santé
Institut du développement et de la santé des enfants et des adolescents
Institut des maladies infectieuses et immunitaires
Institut de l'appareil locomoteur et de l'arthrite
Institut des neurosciences, de la santé mentale et des toxicomanies
Institut de la nutrition, du métabolisme et du diabète
Institut de la santé publique et des populations

Le 30 juin 2016

Docteure Edeltraut Kroger
Centre d'excellence sur le
vieillissement de Québec
CR du CHU de Québec
Hôpital du Saint-Sacrement
1050 Chemin Sainte-Foy
Québec, Québec G1S 4L8

Objet : Partenariats pour l'amélioration du système de santé : Concours 2015-2016, « Health impacts and characteristics of deprescribing interventions in older adults - a systematic review. ».

Docteure Kroger:

Au nom des Instituts de recherche en santé du Canada (IRSC), il me fait plaisir de vous informer que la demande susmentionnée a été retenue pour du financement. Vous recevrez votre autorisation de financement sous peu par la poste.

Étant donné que les IRSC n'informent plus les co-candidats de leur décision, nous vous prions de communiquer le résultat de cette demande aux personnes concernées et à leurs établissements de recherche (s'ils diffèrent du vôtre).

Pour de plus amples renseignements sur le processus d'évaluation, veuillez communiquer avec le Centre de contact des IRSC par courriel à soutien@irsc-cihr.gc.ca ou par téléphone au 1-888-603-4178.

Nous vous souhaitons un franc succès dans vos travaux de recherche.

Veuillez agréer, Docteure Kroger, l'expression de mes meilleurs sentiments.

Nathalie Gendron

Nathalie Gendron, Ph.D.
Gestionnaire, Exécution des concours
Recherche Priorisée
Portefeuille de la recherche, de l’application des connaissances et de l’éthique

431428-201601KRS-KRS-366106-118675-KALA

Canadian Institutes of Health Research
Room 97, 160 Elgin Street, Address locator: 4809A
Ottawa, (Ontario) K1A 0W9 Tel.: (613) 941-2672
Fax (613) 954-1800 **www.cihr-irsc.gc.ca**

Instituts de recherche en santé du Canada
Pièce 97, 160 rue Elgin, Indice de l'adresse: 4809A
Ottawa, (Ontario) K1A 0W9 Tél.: (613) 941-2672
Fax (613) 954-1800 **www.irsc-cihr.gc.ca**

Canada

Institute of Aboriginal Peoples' Health

Institute of Aging

Institute of Cancer Research

Institute of Circulatory and Respiratory Health

Institute of Gender and Health

Institute of Genetics

Institute of Health Services and Policy Research

Institute of Human Development and Child and Youth Health

Institute of Infection and Immunity

Institute of Musculoskeletal Health and Arthritis

Institute of Neurosciences, Mental Health and Addiction

Institute of Nutrition, Metabolism and Diabetes

Institute of Population and Public Health

Institut de la santé des Autochtones

Institut du vieillissement

Institut du cancer

Institut de la santé circulatoire et respiratoire

Institut de la santé des femmes et des hommes

Institut de génétique

Institut des services et des politiques de la santé

Institut du développement et de la santé des enfants et des adolescents

Institut des maladies infectieuses et immunitaires

Institut de l'appareil locomoteur et de l'arthrite

Institut des neurosciences, de la santé mentale et des toxicomanies

Institut de la nutrition, du métabolisme et du diabète

Institut de la santé publique et des populations

Le 30 juin 2016

Docteure Edeltraut Kroger
Centre d'excellence sur le
vieillissement de Québec
CR du CHU de Québec
Hôpital du Saint-Sacrement
1050 Chemin Sainte-Foy
Québec, Québec G1S 4L8

Docteure,

Nous vous félicitons de votre succès au dernier concours de financement des Instituts de recherche en santé du Canada (IRSC).

Vous pouvez en tirer une grande fierté. L'excellence en recherche dépendra toujours de l'inspiration, de la curiosité et de la détermination individuelles. Étant donné la réputation d'excellence dont jouit le milieu de recherche en santé canadien à l'échelle mondiale, nous sommes persuadés que votre travail contribuera à créer de nouvelles connaissances scientifiques qui amélioreront la santé et renforceront le système de soins de santé au profit des Canadiens.

Comme vous le savez, l'évaluation par les pairs est la pierre angulaire de notre système de financement de la recherche. Elle repose sur la bonne volonté de personnes qui acceptent de donner généreusement de leur temps pour évaluer des demandes telles que la vôtre. En tant que chercheur financé par les IRSC, vous pourriez être invité à participer au processus d'évaluation par les pairs, car nous continuons de recruter les chercheurs les plus accomplis, innovateurs et créatifs pour évaluer les propositions de recherche en santé.

Afin d'atteindre les objectifs des IRSC, nous devons aussi continuer à sensibiliser les Canadiens à la valeur de la recherche en santé. C'est pourquoi nous vous encourageons à collaborer avec les IRSC afin de promouvoir votre recherche. Pour vous aider, nous avons élaboré des lignes directrices sur les communications publiques. Vous les trouverez à http://www.cihr-irsc.gc.ca/f/30789.html.

Encore une fois, nous vous offrons nos félicitations et nos meilleurs voeux de succès dans vos travaux de recherche.

Veuillez agréer, Docteure, nos salutations distinguées.

Alain Beaudet, M.D., Ph.D.
Président

431429-201601KRS-KRS-366106-118675-CONGR

President

Canadian Institutes of Health Research
Room 97, 160 Elgin Street, Address locator: 4809A
Ottawa, (Ontario) K1A 0W9 Tel.: (613) 941-2672
Fax (613) 954-1800 **www.cihr-irsc.gc.ca**

Président

Instituts de recherche en santé du Canada
Pièce 97, 160 rue Elgin, Indice de l'adresse: 4809A
Ottawa, (Ontario) K1A 0W9 Tél.: (613) 941-2672
Fax (613) 954-1800 **www.irsc-cihr.gc.ca**

Canada

**Review Type / Type d'évaluation:**	Reviewer 1 / Évaluateur 1
**Name of Applicant / Nom du chercheur:**	Kroger,Edeltraut
**Application No. / Numéro de demande:**	366106
**Agency / Agence:**	CIHR/IRSC
**Competition / Concours:**	Synthesis Grant/Subvention de synthèse
**Committee / Comité:**	Knowledge Synthesis Grant/Subvention sur la Synthèse des Connaissances
**Title / Titre:**	Health impacts and characteristics of deprescribing interventions in older adults - a systematic review.

Concept/Concept

Criterion/Critère: Quality of the Idea/Qualité de l'idée

Rating/Cote: O+

Strengths/Forces: Seniors (65 y+) have an increased risk of adverse health outcomes related to medication use (reasons are physiology, comorbidity, medication interactions)

Over 65% of seniors have at least five prescription medications; 24% of seniors have least one potentially inappropriate medication

Over 65% of adverse drug interactions leading to 10-30% of hospitalizations could be avoided

Among interventions to optimize or reduce seniors' medication load, deprescribing is the discontinuation of chronic medications which are no longer beneficial.

Evidence on the health effects of deprescribing remains inconclusive.

Evidence is also insufficient on which interventions, or elements thereof, are most successful

Weaknesses/Faiblesses: Nothing to mention

Criterion/Critère: Importance of the Idea/Importance de l'idée

Rating/Cote: O+

Strengths/Forces: Support in Quebec, Ontario and at the national level

This systematic review will be imbedded in a knowledge transfer strategy

Weaknesses/Faiblesses: Nothing to mention

Feasibility/Faisabilité

Criterion/Critère: Approach/Approche

Rating/Cote: E++

Strengths/Forces: Search strategies by two scientific librarians, large set of databases + grey literature

Publications in English, French or German.

Two independent reviewers (a research professional and a graduate student). Inter-rater agreement will be assessed

Abstraction forms are provided

Quality assessment of studies (GRADE)

Heterogeneity will be examined, random effects model will be used

Test for publication bias is mentioned

Instead of complete exclusion, studies which cannot be included in a meta-analysis will be included in a narrative synthesis.

Weaknesses/Faiblesses: No time limit although the term deprescribing was proposed in 2003. How can publications prior to 2003 be identified?

Languages other than English, French or German.

No mention that authors could be contacted to obtain missing information.

Since comparison of the interventions are with either usual care, or with different head-to-head interventions, network meta-analysis could be more appropriate than standard meta-analysis.

Outcomes seem too heterogeneous to allow pooling of the results.

Too many subgroup analyses are planned

It is not clear how the quality assessment results will be taken into account in the analyses.

A potential challenge related to the inclusion of cluster randomized trials was not addressed: such trials usually target all the members of a cluster rather than solely those that could benefit from deprescribing.

Criterion/Critère: Expertise, Experience and Resources/Expertise, expérience et ressources

Rating/Cote: O+

Strengths/Forces: The research team is experienced in literature review

Weaknesses/Faiblesses: I believe the applicants should be more involved in article selection and extraction which are crucial phases.

**Review Type / Type d'évaluation:**	Reviewer 2 / Évaluateur 2
**Name of Applicant / Nom du chercheur:**	Kroger,Edeltraut
**Application No. / Numéro de demande:**	366106
**Agency / Agence:**	CIHR/IRSC
**Competition / Concours:**	Synthesis Grant/Subvention de synthèse
**Committee / Comité:**	Knowledge Synthesis Grant/Subvention sur la Synthèse des Connaissances
**Title / Titre:**	Health impacts and characteristics of deprescribing interventions in older adults - a systematic review.

Concept/Concept

Criterion/Critère: Quality of the Idea/Qualité de l'idée

Rating/Cote: O++

Strengths/Forces: This is an excellent proposal from all perspectives (including relevance, timeliness, collaboration, scientific merit, expertise, and integrated KT), in my view.

Weaknesses/Faiblesses: no important weaknesses.

Criterion/Critère: Importance of the Idea/Importance de l'idée

Rating/Cote: O++

Strengths/Forces: A very timely proposal on an important topic for health care.

Weaknesses/Faiblesses: The weakness will be in the studies collected, but that's to be expected.

Feasibility/Faisabilité

Criterion/Critère: Approach/Approche

Rating/Cote: O+

Strengths/Forces: The proposal is impressive in many ways, including integrated KT, strong endorsements from key stakeholders in clinical care (professional organizations, government, academia, practitioners (family medicine, pharmacy, geriatrics).

Weaknesses/Faiblesses: Formal involvement of patients and their personal caregivers appears to be limited. The applicants state that they intend to evaluate end of study KT but no description of the evaluation is provided (no doubt, beyond the scope of the current proposal).

Criterion/Critère: Expertise, Experience and Resources/Expertise, expérience et ressources

Rating/Cote: O++

Strengths/Forces: Excellent and experienced team of experts in the field, methodologists including information specialists, stakeholders.

Weaknesses/Faiblesses: Could be useful to increase involvement of seniors. caregivers, senior's organizations.

**Review Type / Type d'évaluation:**	Reviewer 3 / Évaluateur 3
**Name of Applicant / Nom du chercheur:**	Kroger,Edeltraut
**Application No. / Numéro de demande:**	366106
**Agency / Agence:**	CIHR/IRSC
**Competition / Concours:**	Synthesis Grant/Subvention de synthèse
**Committee / Comité:**	Knowledge Synthesis Grant/Subvention sur la Synthèse des Connaissances
**Title / Titre:**	Health impacts and characteristics of deprescribing interventions in older adults - a systematic review.

Concept/Concept

Criterion/Critère: Quality of the Idea/Qualité de l'idée

Rating/Cote: O++

Strengths/Forces: This application addresses a timely policy about the impacts and characteristics of deprescribing interventions. The investigators highlight specific work that is happening at OPEN that this review can be used to directly inform so it seems to be a timely review to conduct. This section also points to a very strong knowledge user team.

Weaknesses/Faiblesses: A gap in the scope as outlined in this section seems to be that the review will lack information that could speak to the acceptability of the identified interventions from the perspective of different groups in the system (e.g., patients and families as well as physicians). This seems to be a logical complement, and is information that policymakers often ask for but can't find in systematic reviews given limited scope of questions.

Criterion/Critère: Importance of the Idea/Importance de l'idée

Rating/Cote: O++

Strengths/Forces: The investigators make a very strong case for why it is important to pull together the research evidence in this area. The results of the review will be important for informing policy about deprescribing, which is on the agenda of many governments.

Weaknesses/Faiblesses: None noted.

Feasibility/Faisabilité

Criterion/Critère: Approach/Approche

Rating/Cote: O+

Strengths/Forces: This is a superb protocol. Well done. I thought the methods described in section 3 (deriving conclusions and recommendations) and 5 (end-of-grant knowledge exchange) were innovative and will add a lot to the relevance of the findings.

Weaknesses/Faiblesses: As noted previously, it's unfortunate that the scope of the review will be limited to the study designs listed and not include qualitative/acceptability studies that provide additional insight about the interventions that policymakers will want to know about. I realize that this is a Cochrane review, but it could be that through the use of these funds that these types of studies are searched for and included and then written up in a separate manuscript.

Criterion/Critère: Expertise, Experience and Resources/Expertise, expérience et ressources

Rating/Cote: O++

Strengths/Forces: The team brought together for this application is excellent and includes investigators with the content, methodological and KU expertise needed.

Weaknesses/Faiblesses: None noted.

**Review Type / Type d'évaluation:**	Reviewer 4 / Évaluateur 4
**Name of Applicant / Nom du chercheur:**	Kroger,Edeltraut
**Application No. / Numéro de demande:**	366106
**Agency / Agence:**	CIHR/IRSC
**Competition / Concours:**	Synthesis Grant/Subvention de synthèse
**Committee / Comité:**	Knowledge Synthesis Grant/Subvention sur la Synthèse des Connaissances
**Title / Titre:**	Health impacts and characteristics of deprescribing interventions in older adults - a systematic review.

Concept/Concept

Criterion/Critère: Quality of the Idea/Qualité de l'idée

Rating/Cote: E

Strengths/Forces: The overall goal and research questions are well-defined and clear. The potential of these results to impact the health of seniors and the health systems involved in their care is noted. The rationale for this project is sound and logical.

Weaknesses/Faiblesses: It would be helpful to identify and describe planned research and knowledge translation outputs.

Criterion/Critère: Importance of the Idea/Importance de l'idée

Rating/Cote: E

Strengths/Forces: The potential for this project to contribute to seniors health and healthy systems is clearly articulated.

Weaknesses/Faiblesses: N/A

Feasibility/Faisabilité

Criterion/Critère: Approach/Approche

Rating/Cote: E+

Strengths/Forces: The approaches, methods, outputs, timelines, and integrated and end-of-grant KT are appropriate. Potential challenges are considered and mitigation strategies presented.

Weaknesses/Faiblesses: A definition of "chronic medication" would be useful.

Criterion/Critère: Expertise, Experience and Resources/Expertise, expérience et ressources

Rating/Cote: E+

Strengths/Forces: The team appears to have the required expertise and experience.

Weaknesses/Faiblesses: NA

**Review Type/Type d'évaluation:**	SO Notes /Notes de l'agent scientifique
**Name of Applicant/Nom du chercheur:**	Kroger, Edeltraut
**Application No./Numéro de demande:**	366106
**Agency/Agence:**	CIHR/IRSC
**Competition/Concours:**	2016-01-25 Knowledge Synthesis Grant/Subvention sur la synthèse des connaissances
**Committee/Comité:**	Knowledge Synthesis Grant/Subvention sur la Synthèse des Connaissances
**Title/Titre:**	Health impacts and characteristics of deprescribing interventions in older adults - a systematic review.

Assessment/Évaluation:

Votre demande a été examinée par les pairs évaluateurs de la première étape et elle est passée à l'étape d'évaluation finale. Compte tenu de la cote et du rang de votre demande par rapport aux autres demandes évaluées, le comité de l'étape d'évaluation finale a convenu que votre demande était très concurrentielle et qu'elle ne nécessitait pas de discussion approfondie. Par conséquent, l'agent scientifique n'a donc produit aucune note; cependant, les rapports des évaluateurs de la première étape sont disponibles sur RechercheNet. Veuillez consulter l'avis de décision pour plus d'information sur le rang de votre demande. Pour plus d'information sur le processus d'évaluation par les pairs du volet Projet, veuillez consulter Guide d'évaluation par les pairs – concours pilote du volet Projet (http://www.cihr-irsc.gc.ca/f/48645.html).
